# Using Mobile Technology to Improve Bone-Related Lifestyle Risk Factors in Young Women With Low Bone Mineral Density: Feasibility Randomized Controlled Trial

**DOI:** 10.2196/formative.9435

**Published:** 2019-02-25

**Authors:** Asvini Kokila Subasinghe, Suzanne Marie Garland, Alexandra Gorelik, Ilona Tay, John Dennis Wark

**Affiliations:** 1 Infection and Immunity Theme Murdoch Children's Research Institute Parkville Australia; 2 Centre for Women's Infectious Diseases Royal Women's Hospital Parkville Australia; 3 Department of Obstetrics and Gynaecology University of Melbourne Parkville Australia; 4 School of Behavioural and Health Sciences Australian Catholic University Melbourne Australia; 5 Department of Medicine Royal Melbourne Hospital University of Melbourne Parkville Australia; 6 Bone and Mineral Medicine Royal Melbourne Hospital Parkville Australia

**Keywords:** behavior therapy methods, mobile phones, health behavior, primary prevention methods, self-care methods

## Abstract

**Background:**

Poor bone health in adolescent and young adult females is a growing concern. Given the widespread use of mobile phones in this population, mobile health (mHealth) interventions may help improve health behaviors related to bone health in young women.

**Objective:**

The goal of the study was to determine the acceptability and feasibility of an mHealth intervention called Tap4Bone in improving health behaviors associated with the risk of osteoporosis in young women.

**Methods:**

The Tap4Bone mHealth intervention comprised the use of mobile phone apps, short messaging service (text messaging), and Web emails to encourage health behavior changes. The education group received osteoporosis prevention education leaflets. Changes in the bone health–related behaviors exercise, smoking, and calcium intake were assessed. User experiences and acceptance of the app were collected through focus group interviews.

**Results:**

A total of 35 (22 completed, mean age 23.1 [SD 1.8] years) were randomized to either the mobile phone (intervention n=18) or education (control n=17) group. Although there were trends toward improvement in calcium intake, sports activity, and smoking behaviors in the mHealth intervention group compared to the education group, these were not statistically significant.

**Conclusions:**

The Tap4Bone mHealth intervention was shown to be acceptable and feasible in subsets of the participants. The intervention should be improved upon using participant feedback to improve functionality. Findings from this study may aid in the development and modification of health care apps to reduce participant attrition.

## Introduction

The major adverse outcome of poor bone health is osteoporosis with associated fragility fractures [1]. Bone mineral density (BMD) is a clinically useful predictor of fragility fracture risk [2]. During adolescence, young people may be influenced to push boundaries and experiment with risky behaviors that may have an impact on future health trajectories [3]. Many of these lifestyle changes in young women can have an adverse impact on their bone health, increasing the risk of poor outcomes in later life.

There are several identified risk factors that affect peak bone mass accrual and maintenance and may result in the development of this skeletal disorder. Nonmodifiable risk factors include age, female sex, onset of menopause, and genetic predisposition to poor bone health. There are other proposed risk factors that are potentially modifiable such as smoking, high alcohol intake, inadequate physical activity and calcium intake, vitamin D deficiency, and long-term use of corticosteroids [3]. The fact that there is no known cure for osteoporosis and that the risk can be substantially reduced makes it crucial that preventive measures are identified and adopted to reduce the prevalence of this disorder that causes significant morbidity and high economic burden [3].

Findings from prospective longitudinal studies in young female twins (aged 15 to 30 years) highlighted the extent of lifestyle changes in young women [4]. It was found that participation in sports activity decreased with age, with only 23.5% of women aged 27 to 29 years participating in 4 or more hours per week of sports compared to 47.5% of women aged 15 to 17 years [4]. Conversely, sedentary behaviors and smoking habits significantly increased with age, with 14% of women aged 15 to 17 years reporting ever smoking at least one time and by age 27 to 29 years, 51% had smoked [4]. These considerable changes in lifestyle may not have an immediate impact on the skeleton. However, adverse effects are likely to be gradual in onset and accumulate over the life span [3]. Therefore, these adverse lifestyle changes should provide a strong impetus to develop effective interventions to improve risky but modifiable lifestyle factors and improve long-term bone health in young women.

Mobile health (mHealth) is the use of mobile devices to support medical and health practices. Over the past decade, use of mobile phones that include internet and other data packages has grown dramatically. Approximately 81% of the Australian population owns a mobile phone [5]. Of particular interest, the highest ownership and use of mobile phones is found in the 18 to 34 year age group [5]. Approximately 74% to 78% of people in this age group own at least one mobile phone. Therefore, this technology offers a promising tool for health interventions in young people.

In this study, we conducted a 12-week randomized trial to determine whether it is acceptable and feasible to use an mHealth intervention in establishing behaviors known to have favorable influences on bone health, in a sample of young women with relatively low BMD.

## Methods

### Ethics

This study was conducted according to the guidelines laid down in the Declaration of Helsinki and was approved by the Royal Women’s Hospital Human Research Ethics Committee on July 1, 2013.

### Recruitment and Consent

Participants for this study were recruited via telephone from the Young Female Health Initiative (YFHI) study between October 14, 2013, and February 7, 2014. In the YFHI study, a sample of young women were recruited using targeted advertisements on Facebook from May 19 to September 30, 2010, the detailed methodology of which has been reported elsewhere [6].

Clicking on the advertisement directed respondents to secure websites containing more information and where expressions of interest were registered. Prospective participants were then contacted by investigators and consented into the study.

Participation included the completion of a Web-based questionnaire using SurveyMonkey (www.surveymonkey.com) covering demographics, mental health, lifestyle, and reproductive health. Existing participants from the YFHI study who had consented to be contacted for future studies and met the inclusion criteria for this pilot study were contacted by phone to obtain consent. Trained research staff assessed whether the potential participant could be considered a mature minor (if under age 18 years) and that she fully comprehended the purpose, methods, demands, risks, and potential benefits of the study and was capable of giving informed consent to participate. She was required to understand that participation was voluntary and there were no consequences for discontinuing study participation. Staff followed instructions from the Medical Practitioners Board of Victoria “Consent for Treatment and Confidentiality in Young People” on how to define a mature and competent young person.

### Bone Density Assessment

In the YFHI study, participants had their BMD at the total body, lumbar spine, and hip measured at the Royal Melbourne Hospital using dual-energy x-ray absorptiometry (DXA) (QDR 4500A densitometer, Hologic Inc). Participants who had relatively low (z score ≤–0.5 at the total hip, femoral neck, or lumbar) or otherwise abnormal (z score <–1.0) bone density results were informed via postal letter including advice on appropriate follow-up. A copy of the participant’s BMD report was enclosed with the letter. Participants were advised to discuss the result and any necessary further management with their usual doctor. Only participants who met the bone density cutoff values specified below were contacted for this study.

### Inclusion and Exclusion Criteria

Inclusion criteria were met if individuals were female aged 16 to 25 years, residing in Victoria, Australia, and having a BMD z score ≤–0.5 at the total hip, femoral neck, or lumbar spine region from DXA. According to the World Health Organization, a z score unit represents 1 standard deviation above or below the age- and sex-matched mean BMD for a specific skeletal site, and a z score higher than –1.0 (equivalent to a T-score of –1.0 in young adults as defined by the World Health Organization) is considered normal [7]. However, for inclusion in this study we wished to define a sample with relatively low, but not necessarily abnormal, BMD. It was considered that such a group may be motivated to undertake a lifestyle-oriented intervention to improve their bone health. Selecting this cutoff also enabled us to achieve the desired sample size.

Participants were excluded if they had a BMD z score >–0.5 at all measured sites, current or history of any significant medical conditions, including eating disorders, or were pregnant or breastfeeding. Individuals were also excluded if they did not own iPhones or Android phones or were not willing to use them for the study.

### Randomization Detail

Participants were randomized 1:1 to either the education or mHealth intervention group after completing a baseline questionnaire on current calcium intake, physical activity levels, and smoking habits if applicable. Participants were not aware of which intervention was of interest and which one was the comparator.

### Randomization Sequence Generation

An independent researcher generated the randomization sequence and provided the principal investigator with each treatment allocation. The allocations were sealed in white opaque envelopes with only the randomization sequence numbers written on the front of the sealed envelopes. This ensured that the researchers conducting the study were blinded to the treatment groups to which each patient was randomized.

Participants were randomized into the 2 intervention groups by stratified block randomization in blocks of 2, 4, 6, and 8. Participants were stratified by tertiary education (yes/no) and z score.

- No tertiary education and z score ≤–0.5 to –1.0 at any region (total hip, lumbar spine, or femoral neck)
- No tertiary education and z score <–1.0 to –2.5 at any region (total hip, lumbar spine, or femoral neck)
- Currently completing or completed tertiary education and z score ≤–0.5 to –1.0 at any region (total hip, lumbar spine, or femoral neck)
- Currently completing or completed tertiary education and z score <–1.0 to –2.5 at any of region (total hip, lumbar spine, or femoral neck)

### Interventions

#### Education Intervention

Participants were emailed educational leaflets on osteoporosis prevention downloaded from the US National Institutes of Health website [8].

Participants were then asked to complete an assessment of understanding that evaluated their knowledge of bone health and osteoporosis. The assessment comprised 10 true/false questions based on the educational leaflets. Participants were required to answer 6 out of the 10 questions correctly to proceed with the study.

#### mHealth Intervention

The Tap4Bone mHealth intervention incliuded using mobile phone apps and sending and receiving short messaging service (SMS) text messages and web emails. In addition to the educational leaflets, participants in the mHealth group received suggestions for other mobile phone apps to be used as behavior change techniques (BCTs), including the following:

- Self-monitoring of diet and exercise using mobile phone app
- Encouragement and motivations from text messages
- Goal-setting and checking forms via the survey tool Limesurvey (www.limesurvey.org)
- Progress feedback via emails

Additionally, they were emailed a guidance document on how to download and use other mobile phone apps. Participants in the mHealth group also received 5 questions in the assessment to determine their understanding of the guidance document and how to properly use the mobile phone apps.

#### Other Mobile Phone Apps

Participants were asked to download and use 3 recommended mobile phone apps: MyFitnessPal, Nike Training Club, and QuitBuddy. MyFitnessPal is a free calorie counter app that helps people track their diet and exercise. Nike Training Club is a free app comprising more than 100 full-body workouts. This app also tracks the overall amount of workout time completed by the users but it does not take into account when the exercise was completed. Therefore, for the purpose of tracking and providing feedback on participant progress, we encouraged them to track their physical activities in MyFitnessPal. QuitBuddy is a smoking cessation interactive app developed by the Australian National Preventive Health Agency to help smoking cessation efforts.

Participants also received motivational text messages that were sent out randomly using an automatic system (SMS Broadcast) during the study. Various text messages providing suggestions on how to change participant behaviors or giving them encouragement were sent. Suggestions included tips on how to increase dietary calcium intake and improve physical activity. Participants received one text daily for the first 3 weeks, and subsequently, 3 texts per week for the next 9 weeks.

Participants were also asked to set behavior goals on day 1 of week 1, week 3, week 6, and week 9. They could set dietary goals, exercise goals, or smoking cessation goals or a combination of all 3 types. The behavioral goals they selected were emailed to them to remind them of the goals they aimed to attain. At every goal-setting time point, participants were asked to check against the goals that they had achieved during the previous 3 weeks.

Finally, participants received biweekly feedback reports on their behavior change progress. We used the diet and exercise diaries entered by them in MyFitnessPal and smoking habits provided via SMS to provide individual progress feedback on their calcium intake and exercise progress. The data were collated and sent via email to the participants for personal monitoring of their progress.

### Exit Survey

Participants in both groups were asked to complete an exit survey at the end of week 12. The purpose of this survey was to find out whether they had self-initiated changes to their lifestyle in response to the intervention they received. In addition, participants were asked for their initial perceptions of a mobile phone app to help improve young women’s bone health and the potential for implementation in their daily lives.

Participants in the mHealth intervention group also completed a usability questionnaire in which they were asked about their experience with the mobile phone intervention. The usability questionnaire covered the use of the different components such as the app, goal-setting, motivational text messages, and progress reports as well as the overall perception of the future use of a bone health mobile phone app. See Multimedia Appendix 1 for an outline of all study activities.

### mHealth Focus Group

Participants from the mHealth group who completed the week 12 questionnaires and exit survey were invited to a focus group. The focus session of 30 minutes’ duration was conducted within 2 months after completion of the week 12 questionnaires and exit survey.

One investigator conducted a qualitative semistructured interview using responses from the mobile focus group to explore participant perceptions of Tap4Bone and whether it might be implemented into their everyday lives. The topics covered during the interview included (1) how the intervention was used, (2) what components were useful, and (3) how the intervention affected their behaviors. The focus groups were conducted using a free mobile phone app called WhatsApp Messenger as described elsewhere [9]. The semistructured mobile focus group interview data were analyzed the same way as described in the Calci-app study [9].

### Study Outcomes and Measurements

#### Primary Outcome

The primary outcomes was the percentage and direction of change (either a positive or negative behavioral change) in individual health behaviors at week 12.

- Number of cigarettes smoked per day
- Daily calcium intake (measured in milligrams per day)
- Weekly physical activity score (measured in metabolic equivalent of task: minutes per week)

An overall cumulative risk factor score will be reported from the net percentage change, which is calculated by combining the percentage change in each individual risk factor.

#### Secondary Outcome

The secondary outcome was the feasibility of the intervention in young women. We measured the feasibility of the mobile phone–based intervention using study completion rate and responses to the usability questionnaire and semistructured interview.

### Sample Size Calculation

The sample size was calculated based on the anticipated number of participants from the YFHI study who would fulfill the required bone density parameters. We estimated that 150 participants would have completed the YFHI study’s site visits from October 2012 to November 2013. From this sample, we estimated that 33% (with a mean of 0 and standard deviation of –2.5 in a normal distribution) would have z scores between –0.5 and –2.5. Taking into consideration that 20% of the recruited participants might decline participation, withdraw consent, or be lost to follow-up, we aimed to recruit 40 participants in total for this study.

A target of 40 to 50 participants was determined to be an appropriate size to determine the effect of the intervention. This number was not powered to measure outcomes at 12 weeks as this is an exploratory study. Post hoc power analysis will be done to determine the study’s statistical power and effect size for further potential studies.

### Statistical Analysis

Statistical analysis was performed with Stata version 15 (StataCorp LLC). Descriptive statistics were used to summarize participant experiences with results reported as frequency and percentage. A Student *t* test was used to investigate significant differences between groups. Thematic analysis was undertaken to analyze qualitative data [9].

## Results

### General

**Figure 1:**
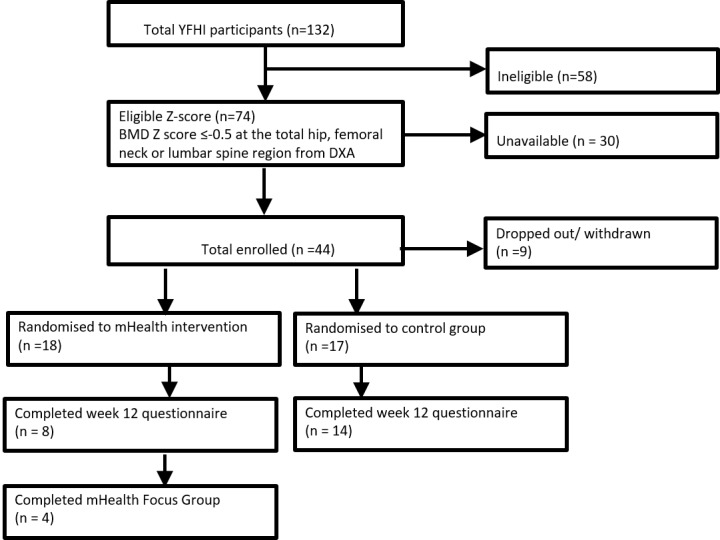
Flowchart for recruitment of participants. YFHI: Young Female Health Initiative, mHealth: mobile health, BMD: bone mineral density, DXA: dual-energy x-ray absorptiometry.

Of the 74 young women who met the inclusion criteria, 44 agreed to participate, while the other 30 subjects were either not contactable or declined participation (Figure 1).

Of the 44 participants recruited into Tap4Bone, 9 withdrew prior to randomization and 35 were randomized into either mobile phone (intervention n=18) or education (control n=17) groups. Reasons given for participant withdrawal included lack of time and being away overseas. Participants who could not be contacted after 3 attempts via different modes of communication (phone, email, or SMS) were considered lost to follow-up.

### Efficacy of Intervention in Overall Study

We were unable to detect significant differences between a change in calcium and lifestyle behaviors for participants in each group across the 12 weeks (Table 1).

**Table 1:** Demographic and lifestyle characteristics of participants (N=35).

Characteristics		mHealth intervention (n=18)	Control group (n=17)	*P* value
Age in years, mean (SD)		23.3 (2)	22.3 (2)	.08
Body mass index (kg/m^2^), mean (SD)		22.6 (3)	24.1 (5)	.28
**BMD^a^** **category, n (%)**				
	Normal (–0.5 to –1.0)	8 (44)	6 (35)	.58
	Osteopenia (–1.0 to –2.5)	10 (56)	11 (65)	
Calcium intake (mg/d), mean (SD)		618.5 (472.2)	645.0 (309.2)	.85
Physical activity (hours per week), mean (SD)		12.5 (15)	15.56 (19)	.60
Smoking (pack-years), mean (SD)^b^		2.8 (2)	1.5 (1)	.46
Change in calcium intake over 12 weeks, %^c^		3.34	0.17	.29
Change in physical activity over 12 weeks, %^c^		0.41	0.07	.42
Change in smoking over 12 weeks, %^d^		0	–0.74	—
Net change in risk factors (%)		3.76 (9)	0.24 (1)	.25

^a^BMD: bone mineral density.

^b^Data available for 4 participants from the mHealth intervention group and 6 participants from the control group.

^c^Data available for 7 participants from the mHealth intervention group and 9 participants from the control group.

^d^Data available for 4 participants from the control group.

### Qualitative User Experience

#### Thematic Analysis

Thematic analysis of the focus group interviews (n=4) was used to summarize mHealth participant experiences with Tap4Bone and the feasibility of such an intervention in young women. Four distinct themes were identified: (1) need to target the right audience, (2) increased lifestyle awareness, (3) experience with integrating BCTs into lifestyle, and (4) need to maintain focus on bone health.

#### Need to Target the Right Audience

When asked how other young women would perceive such an intervention, all participants identified that targeting the correct audience was the first and most critical step in the intervention uptake. Participants in the study proposed that young women who know that they have low bone density would be more interested in the intervention than those who have normal bone density and those who do not know of their bone density status. Besides targeting the intervention at young women with low bone density, one participant expressed that the information also needs to be relevant for young women in this age group.

Absolutely. If your bone density results were perfect you would continue doing what you're doing. If you know they're bad you'd happily download the app to improve your own health.

Participant A

And knowing your bone density would be crucial to your interest in an app.

Participant D

Telling other female friends about this study, none were particularly interested.

Participant C

#### Increased Lifestyle Awareness

The intervention was successful in raising daily awareness of participant lifestyle behaviors. However, implementation of changes in their lifestyles was restricted mostly to increasing dietary calcium intake and not physical activity. One of the likely reasons could be that the exercise app contained irrelevant workouts and lacked a reward and recognition system to motivate participants. Moreover, 3 out of 4 participants had a regular exercise routine and were already using other exercise apps (such as MapMyRun and Lorna Jane) before they started Tap4Bone. Almost all felt that monitoring their diet and comparing it with recommended daily calcium intake had significantly helped them increase their dietary calcium intake.

I suppose that having reminders, and the requirements to record them, meant that you were aware all the time.

Participant B

I am more aware of my calcium intake as well, because of the study.

Participant C

However, it was unanimously agreed that the heightened lifestyle awareness, particularly from keeping a food diary, could be confronting and it inevitably stimulated bad eating thoughts. They acknowledged that young women put a lot of pressure upon themselves to look good and are often worried about body image and weight control. As the app also tracked calories, participants felt that they eventually became used to caloric counting.

If it's any interest to you, I have actually stopped using MyFitnessPal as I felt I was becoming quite consumed by my kilojoule intake which was frustrating!

Participant D

On a positive note, half of the participants felt that the intervention had helped ignite a focus on health and fitness and also shifted their focus on exercise for weight loss to exercise for preventative health, in this case, for building bone density.

I was already using MyFitnessPal before starting the program. I was using it for weight loss it was good to help track calcium consumption.

Participant C

I think that young women have a lot of pressure on them to eat well and exercise to look good, but providing them with information about long term benefits of eating a lot of calcium and encouraging them to exercise for a preventative measure would be well received. This was one thing I got from this study, I re-jigged my concept of exercise as being about healthy bones and healthy body rather than about weight loss.

Participant D

#### Experience With Integrating Behavior Change Techniques Into Lifestyle

Participants did not find the respective BCTs (texts, goal setting, or progress feedback) particularly useful on their own. However, they found the prospect of having an all-in-one app that connects each of the BCTs in a relational way very favorable. For example, text messages could be personalized with the goals they have set, instead of random motivational texts that most of them found artificial and patronizing. The texta could also be more informative and serve as reminders to help them attain their goals.

Although, also a record of goals set on the actual App would be helpful. It needed to be more centralized because there were components all over the place, email, MyFitnessPal, Nike, the online survey etc.

Participant B

If the texts could have been more focused on your individual goals I think that would have been better. So if you said you would have 1 slice of cheese a day a text daily that said “have you had your cheese slice today?” Might have been more useful.

Participant A

The main priorities participants looked for in a bone health intervention app were motivation, relevance, and usefulness. Also, the extreme preferences for text messages (ranging from very negative to very positive) meant that participants preferred a flexible app in which they could adjust their preferences. The need for flexibility was also reflected in their request for more personalized and practical goals that they could implement into their individual lifestyle. Therefore, although the BCTs were not useful in isolation, integrating them into a holistic app was well received.

The text message reminders were great, made you really think about what you needed to eat/drink each day.

Participant A

Sorry to say I would be happy not to receive them at all. I think that’s more specific to me though.

Participant C

A bit more freedom in the goal setting, too, would help. For example, I don’t like drinking whole glasses of milk, and I don’t like hard cheese, so those weren’t things I was going to ever do.

Participant B

#### Need to Maintain Focus on Bone Health

An interesting observation raised by one participant was that young women are willing to overlook bone health for body image reasons and weight management. As a result, participants felt that they became distracted with the caloric measurements in MyFitnessPal while monitoring their calcium intake.

Also some advice that tied between exercise and calcium. I’ve found a lot of personal trainers get you to stray away from lots of cheese and milk, which confuses the hell out of me because I know you need those things for calcium.

Participant A

Participants felt that the intervention seemed to continue for too long; consequently, they lost focus on the overall goal of building bone density.

Yeah it did go for a long time and I have to admit I lost site of the overall goal of building bone density.

Participant D

Agreed D. By the end of it I was really more focused on exercise than bone density.

Participant A

With me I was more concerned with KJ intake.

Participant C

Hence, it was quite possible that the combination of distraction from caloric counting and lack of visible progress (ie, bone density change measurements) contributed to the shift in focus from bone density building to caloric management.

To bring the focus back to bone health, participants wished that there were more bone-related information and emphasis on relevant consequences of suboptimal bone health. However, it is a challenge to shift the focus on bone density and sustain it since bone-related consequences are only visible many decades later.

I must admit the study got me onto a health and fitness kick, but not focused on calcium. I think more information about calcium intake throughout the study would have helped. More case studies, more Q&A’s, facts for us to read along the way to remind us WHY we were taking part and the benefits to us.

Participant A

I actually learned I was extremely vitamin D deficient and it wasn't calcium intake that was my problem, so maybe some more info about how the body uses calcium to build bones and reminders about that rather than just simply about eating cheese.

Participant D

## Discussion

### Principal Findings

In this study, the acceptability and feasibility of an mHealth intervention, Tap4Bone, was trialed in young women with below average bone health measures, with some features working well within subsets of the participants. Participants perceived value in the mHealth intervention and did not indicate that it caused them additional burden. When asked about their interest in a mobile phone app with all the Tap4Bone functions, 3 out of 4 users said that they would be likely to use it in the future to improve their bone health. Importantly, participants reported that the prospect of having an all-in-one app that connects each of the BCTs in a relational way was optimal. Participants also believed that the intervention would be more useful with the involvement of health professionals. The critical function of health care delivery would be to provide support and encouragement to patients to help them achieve healthier behaviors and self-manage chronic illnesses. However, support and encouragement rendered to patients are limited within existing health care infrastructures and even traditional informational media (brochures, posters, etc). This would be where the far-reaching capabilities of mobile technologies can be tapped into to allow low-cost interventions and support interactivity, hence allowing patients to “obtain extra help when needed” [10-12].

Although there are many dietary mobile phone apps available commercially, to our knowledge this is the first study investigating the feasibility of using mobile technology to improve calcium intake and other bone-related lifestyle behaviors in young women. Further, for this study we selected suitable BCTs taken from a taxonomy [13] that has been proven to be effective in lifestyle behavior change interventions. Previous meta-analyses have identified a specific set of BCTs that have been shown to be effective in interventions to promote healthy eating, physical activity, and smoking cessation, most of which have been included in this study [14,15].

### Limitations

This study had several limitations. While the sample was randomly recruited through Facebook and was representative of the general population in the same age range in terms of socioeconomic factors and country of birth, those with greater education levels and older participants were slightly overrepresented [6]. Also, in the absence of a placebo control group that receives no active intervention, the ability to uncover the true effects of the mobile technology intervention was limited. However, in other randomized controlled trials in which education was used alone, investigators found no changes in lifestyle behaviors [16]. Therefore, we considered that the education group in our study provided a reasonable control for comparison with the mHealth group. We also had a number of participant withdrawals and a small sample size which may have reduced the likelihood of our detecting significant differences in behaviors between the two groups. However, it should be noted that the primary aim of this study was to evaluate the feasibility of implementing an mHealth intervention. Last, a longer period of follow-up would be needed to ascertain long-term adherence to the intervention and the effects of persistent lifestyle changes on BMD outcomes.

As we have demonstrated that an mHealth intervention targeted at young women with below average bone health measures has shown some aspects of acceptability, the next phase of this study will involve taking on board participant feedback and creating Tap4Bone as one mobile phone app comprising the different online methods of communication we have discussed in this paper. By trialing this stand-alone mobile app with a larger cohort of young women, we will also then have adequate power to investigate the changes in behaviors we looked at in a preliminary fashion in this study.

### Conclusion

We have demonstrated that an mHealth intervention is an acceptable and feasible method of engaging young women with suboptimal bone health. To further improve the functionality and purpose of Tap4Bone, we will use constructive feedback provided by participants to develop a single mHealth app in which input from health professionals will be integrated.

## Appendix

Multimedia Appendix 1 Study activities for participants.

## Abbreviations

BCT: behavior change technique
BMD: bone mineral density
DXA: dual-energy x-ray absorptiometry
SMS: short message service
YFHI: Young Female Health Initiative

## References

[1] Ferrari S, Bianchi ML, Eisman JA, Foldes AJ, Adami S, Wahl DA, Stepan JJ, de Vernejoul M, Kaufman J, IOF Committee of Scientific Advisors Working Group on Osteoporosis Pathophysiology (2012). Osteoporosis in young adults: pathophysiology, diagnosis, and management. Osteoporos Int.

[2] Divittorio G, Jackson KL, Chindalore VL, Welker W, Walker JB (2006). Examining the relationship between bone mineral density and fracture risk reduction during pharmacologic treatment of osteoporosis. Pharmacotherapy.

[3] Australian Institute of Health and Welfare.

[4] Christie J, Nowson C, Garland S, Wark J, Burckhardt P, Dawson-Hughes B, Weaver C (2013). Emerging nutritional and lifestyle risk factors for bone health in young women: a mixed longitudinal twin study. Nutritional Influences on Bone Health.

[5] Sensis Social Media Report 2017: Chapter 1—Australians and social media.

[6] Fenner Y, Garland S, Moore E, Jayasinghe Y, Fletcher A, Tabrizi SN, Gunasekaran B, Wark JD (2012). Web-based recruiting for health research using a social networking site: an exploratory study. J Med Internet Res.

[7] (2004). WHO Scientific Group on the Assessment of Osteoporosis at the Primary Health Care Level: summary meeting report.

[8] National Institutes of Health.

[9] Tay I, Garland S, Gorelik A, Wark JD (2017). Development and testing of a mobile phone app for self-monitoring of calcium intake in young women. JMIR Mhealth Uhealth.

[10] Free C, Phillips G, Galli L, Watson L, Felix L, Edwards P, Patel V, Haines A (2013). The effectiveness of mobile-health technology-based health behaviour change or disease management interventions for health care consumers: a systematic review. PLoS Med.

[11] Free C, Whittaker R, Knight R, Abramsky T, Rodgers A, Roberts I (2009). Txt2stop: a pilot randomised controlled trial of mobile phone-based smoking cessation support. Tob Control.

[12] Rodgers A, Corbett T, Bramley D, Riddell T, Wills M, Lin R, Jones M (2005). Do u smoke after txt? Results of a randomised trial of smoking cessation using mobile phone text messaging. Tob Control.

[13] Abraham C, Michie S (2008). A taxonomy of behavior change techniques used in interventions. Health Psychol.

[14] Michie S, Abraham C, Whittington C, McAteer J, Gupta S (2009). Effective techniques in healthy eating and physical activity interventions: a meta-regression. Health Psychol.

[15] West R, Walia A, Hyder N, Shahab L, Michie S (2010). Behavior change techniques used by the English Stop Smoking Services and their associations with short-term quit outcomes. Nicotine Tob Res.

[16] Blalock S, Currey S, DeVellis R, DeVellis B, Giorgino K, Anderson J, Dooley M, Gold D (2000). Effects of educational materials concerning osteoporosis on women's knowledge, beliefs, and behavior. Am J Health Promot.

