# Supplementary material for: Using Mobile Technology to Improve Bone-Related Lifestyle Risk Factors in Young Women With Low Bone Mineral Density: Feasibility Randomized Controlled Trial
Source: JMIR Form Res. 2019 Feb 25;3(1):e9435. doi: 10.2196/formative.9435 (PMC6409511; doi:10.2196/formative.9435)
Supplement: Multimedia Appendix 1 [file formative_v3i1e9435_app1.docx]

Multimedia Appendix 1. Study activities for participants

| Activity | Screening | Post-screening | Day 1 of W1 | W2 | W3 | W4 | W5 | W6 | W7 | W8 | W9 | W10 | W11 | W12 |  |
| --- | --- | --- | --- | --- | --- | --- | --- | --- | --- | --- | --- | --- | --- | --- | --- |
| Informed consent | X |  |  |  |  |  |  |  |  |  |  |  |  |  |  |
| Eligibility check | X |  |  |  |  |  |  |  |  |  |  |  |  |  |  |
| Educational leaflets |  | X |  |  |  |  |  |  |  |  |  |  |  |  |  |
| Assessment of understanding |  | X |  |  |  |  |  |  |  |  |  |  |  |  |  |
| Training materials^1^ | X | X |  |  |  |  |  |  |  |  |  |  |  |  |  |
| Baseline questionnaire | X |  |  |  |  |  |  |  |  |  |  |  |  |  |  |
| Follow-up questionnaire |  |  |  |  |  |  |  | X |  |  |  |  |  |  |  |
| Final questionnaire |  |  |  |  |  |  |  |  |  |  |  |  |  | X |  |
| Study information and OP information SMS^2^ |  |  | X |  | X |  | X |  | X |  | X |  | X |  |  |
| Mobile phone intervention^1^ |  |  | X | X | X | X | X | X | X | X | X | X | X | X |  |
| Goal-setting and goals check^1^ |  |  | X |  | X |  |  | X |  |  | X |  |  | X^3^ |  |
| Personalised SMS^1^ |  |  | X | X | X | X | X | X | X | X | X | X | X | X |  |
| Progress Report^1^ |  |  |  | X |  | X |  | X |  | X |  | X |  | X |  |
| 1- Applicable to only participants in the intervention group | | | | | | | | | | | | | | | |
| 2- Applicable to only participants in the control group  3- At Week 12, only goal checks will be performed | | | | | | | | | | | | | | | |
